# Construction and verification of a risk factor prediction model for neonatal severe pneumonia

**DOI:** 10.3389/fmed.2025.1536705

**Published:** 2025-06-02

**Authors:** Weihua Gong, Kaijie Gao, Jiajia Ni, Ying Shi, Zhiming Shan, Hongqi Sun, Shanshan Wang, Jiangtao Xu, Junmei Yang

**Affiliations:** ^1^Department of Clinical Laboratory, Children’s Hospital Affiliated to Zhengzhou University, Zhengzhou Key Laboratory of Children’s Infection and Immunity, Zhengzhou, Henan, China; ^2^Department of Detection and Diagnosis Technology Research, Guangzhou National Laboratory, Guangzhou, China; ^3^Department of Neonatal Disease Screening, The Third Affiliated Hospital of Zhengzhou University, Zhengzhou, China

**Keywords:** neonatal, severe pneumonia, predictive model, nomogram, risk factor

## Abstract

**Objective:**

To construct and validate a risk factor prediction model for neonatal severe pneumonia.

**Methods:**

This study collected data from newborns diagnosed with pneumonia in Children’s Hospital Affiliated to Zhengzhou University. A total of 652 newborns were included. Risk factors were identified using Least Absolute Selection and Shrinkage Operator (LASSO) regression and logistic regression analysis. The nomogram was used to construct a prediction model. The effectiveness of the model was evaluated using calibration curve, receiver operating characteristic (ROC) curve, and decision curve analysis (DCA).

**Results:**

Out of 652 newborns, 186 (29%) were diagnosed with severe pneumonia. The patients were randomly divided into a training set (*n* = 554) and a testing set (*n* = 98) in a ratio of 85:15. A total of 30 indicators were analyzed. Respiratory rate (OR = 1.058, 95% CI: 1.035–1.081), weight (OR = 0.483, 95% CI: 0.340–0.686), C-reactive protein (CRP) (OR = 1.142, 95% CI: 1.028–1.268), neutrophil (NEU) (OR = 1.384, 95% CI: 1.232–1.555), hemoglobin (HGB) (OR = 0.989, 95% CI: 0.979–0.999), uric acid (UA) (OR = 1.006, 95% CI: 1.002–1.010), and blood urea nitrogen (BUN) (OR = 1.230, 95% CI: 1.058–1.431) were identified as independent risk factors for neonatal severe pneumonia. The calibration curve showed significant agreement. The area under the ROC curve (AUC) was 0.884 (95% CI: 0.852–0.916) for the training set, and 0.835 (95% CI: 0.747–0.922) for the testing set. DCA demonstrated good predictive properties.

**Conclusion:**

The prediction model based on respiratory rate, weight, CRP, NEU, HGB, UA, and BUN has shown promising predictive value in distinguishing between mild to moderate pneumonia and severe pneumonia in neonates.

## Introduction

Neonatal pneumonia is a common respiratory tract infection that causes significant morbidity and mortality worldwide. It is estimated that between 152,000 and 490,000 infants under the age of one die each year due to this condition (1). Compared to children and adults, newborns have limited ability to defend against lung infections, making them more susceptible to developing severe pneumonia. Data shows that the majority of pneumonia-related deaths are caused by severe cases of the illness (2, 3). Severe pneumonia is characterized by a sudden onset, rapid progression, and high mortality rate, often leading to multiple organ dysfunction. Moreover, it is frequently accompanied by hemodynamic instability, which may require mechanical ventilation and can result in increased mortality and complications both within and outside of the lungs (4). Therefore, it is crucial to identify risk factors early on and alert clinicians to implement interventions as soon as possible.

We typically base our diagnosis on a combination of patient physical examination findings, radiological evidence, and laboratory data. According to the United States Centers for Disease Control and Prevention (CDC), the criteria for diagnosing pneumonia in infants under 1 year of age include radiographic evidence of persistent lung consolidation, cavitation, or pleural effusion, worsening gas exchange, and at least three clinical and/or laboratory evidences (Supplementary Table S1) (5–7). However, diagnosing pneumonia during the neonatal period is challenging. Neonatal pneumonia progresses rapidly, and its clinical manifestations are often atypical, making it difficult to diagnoses. This is because the early clinical symptoms of infantile pneumonia, such as cough, fever, shortness of breath, and dyspnea, are often non-specific (8). Additionally, the absence of distinctive physical signs makes early diagnosis complicated. While certain guidelines can assist clinicians in decision-making, there is currently no effective standard for assessing the severity of neonatal pneumonia. Therefore, identifying of high-risk individuals is crucial for preventing severe neonatal pneumonia and its associated adverse outcomes. However, there have been limited attempts to establish a diagnostic model for assessing the severity of neonatal pneumonia. The purpose of this study was to develop an objective and accurate prediction model for risk factors of neonatal severe pneumonia, using clinical and laboratory data, and to evaluate the model’s predictive accuracy. This will provide valuable insights for early screening and intervention in clinical medicine.

## Materials and methods

### Study design and setting

**FIGURE 1:**
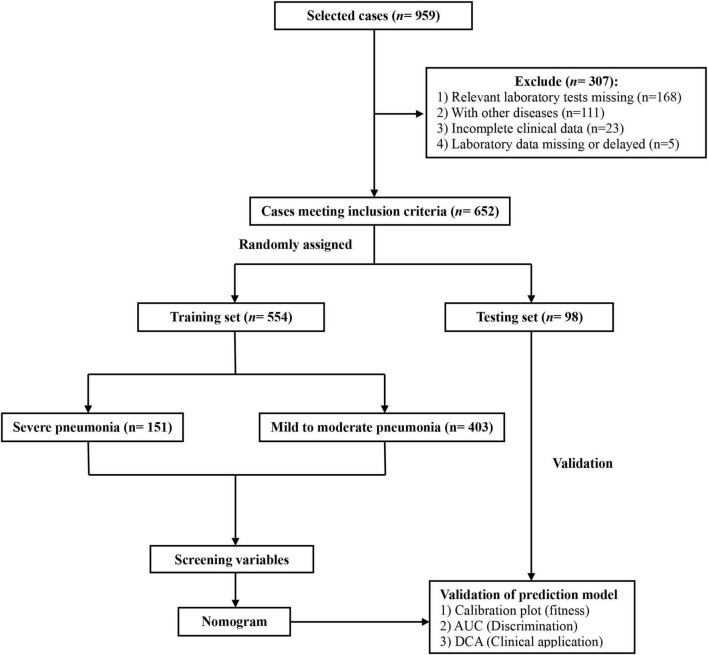
The flow diagram for developing and validating the prediction model for neonatal severe pneumonia.

This study retrospectively included newborns diagnosed with pneumonia who were admitted to the Children’s Hospital Affiliated to Zhengzhou University from January 2019 to December 2022. The inclusion criteria were: (1) Meeting the diagnostic criteria for mild to moderate or severe neonatal pneumonia (3, 9); (2) Undergoing standardized initial laboratory examination within 48 h of the first admission, with all required examinations results meeting the diagnostic criteria; (3) Age 1–28 days; (4) Having complete clinical data collected. The exclusion criteria were: (1) Neonates who did not undergo relevant laboratory tests; (2) Neonates with other diseases, such as congenital pulmonary dysplasia, congenital kidney disease, congenital heart disease, major congenital malformations, hematological diseases, cancer, and malnutrition; (3) Neonates without complete clinical data; (4) Neonates with missing or delayed ( > 48 h) laboratory data. A total of 652 neonatal pneumonia patients were ultimately included. Based on the 85:15 randomization ratio, they were divided into a training set (*n* = 554) and a testing set (*n* = 98). The flow diagram for developing and validating the prediction model is shown in Figure 1. This research protocol followed the Declaration of Helsinki and was approved by the Ethics Review Board of the Children’s Hospital Affiliated to Zhengzhou University (No. 2022-K-L059), which waived individual informed consent as the study used anonymized data without intervention. A prerequisite for the Committee to waive the informed consent is that when the patients were admitted to the hospital, a notice had been signed from their guardians (usually their parents) agreeing to use their clinical data and imaging data for scientific research. Patients whose guardians had previously declared refusal to participate in research were excluded. All data were de-identified prior to analysis to protect confidentiality.

### Definition and data collection

In this study, all children who were treated at the Children’s Hospital Affiliated to Zhengzhou University were included in the case system. The diagnostic criteria for mild to moderate pneumonia and severe pneumonia were based on the severity assessment of community-acquired pneumonia (CAP) in children by the British Thoracic Society (10). Mild to moderate pneumonia in infants is characterized by a temperature below 38.5°C, a respiratory rate below 50 breaths per minute, mild inspiratory depression of the chest wall, and a normal diet. Severe pneumonia is characterized by a temperature above 38.5°C, a respiratory rate above 70 breaths per minute, moderate to severe inspiratory depression of the chest wall, nasal alar fanning, cyanosis, intermittent apnea, moaning, refusal to eat, tachycardia, and capillary congestion lasting for more than 2 s. After enrollment, the following clinical data were collected within 24 h of admission: gender, age, temperature, heart rate, respiratory rate, and weight. Simultaneously, laboratory examination data were also collected, including procalcitonin (PCT), C-reactive protein (CRP), white blood cell (WBC) count, neutrophil (NEU) count, lymphocyte (LYM) count, monocyte (MON) count, basophil (BAS) count, eosinophil (EOS) count, platelet (PLT) count, red blood cell (RBC) count, hematocrit (HCT), hemoglobin (HGB) level, red blood cell distribution width (RDW), total protein (TP) level, albumin (ALB) level, globulin (GLOB) level, alkaline phosphatase (ALP) level, alanine aminotransferase (ALT) level, aspartate aminotransferase (AST) level, gamma-glutamyl transferase (GGT) level, creatinine (CREA) level, uric acid (UA) level, and blood urea nitrogen (BUN) level. PCT levels were measured using an electrochemiluminescence assay (Elecsys^®^ BRAHMS PCT kit, Roche Diagnostic, Rotkreuz, Switzerland) on a Cobas^®^ 8,000 modular analyzer (Roche Diagnostic, Rotkreuz, Switzerland). CRP was measured using a latex-enhanced immunoturbidimetric assay (Ultrasensitive CRP kit, Upper Bio-Tech, Shanghai, China) on an UPPER analyzer (Upper Bio-Tech, Shanghai, China). WBC count, NEU count, LYM count, MON count, BAS count, EOS count, PLT count, RBC count, HCT, HGB level, and RDW were measured by an automated blood cell counter (Sysmex Corporation, Kobe, Japan). The levels of TP, ALB, GLOB, ALP, ALT, AST, GGT, CREA, UA, and BUN were measured using an automatic biochemistry analyzer (AU5800 Clinical Chemistry Analyzers, Beckman Coulter, California) and a conventional clinical analytical method.

### Model development and validation

Despite data is commonly randomly split into 70% for training and 30% for testing, or 80% for training and 20% for testing to machine learning model (11), in this study, because the sample size was too small, the dataset was randomly divided into a training set and a testing set in a ratio of 85:15. To ensure randomization and reproducibility of the sampling, we set a random seed. The training set was used to analyze the risk factors of neonatal severe pneumonia and establish a predictive model. The effectiveness and clinical application value of the model was evaluated using the testing set. We first employed Least Absolute Shrinkage and Selection Operator (LASSO) regression analysis to construct and validate the prediction model. The potential risk factors were selected based on their non-zero coefficients. These factors were then incorporated into a multivariate logistic regression analysis, and independent risk factors with a *p*-value < 0.05 were included in the nomogram model. The nomogram was used to illustrate the risk factors. The accuracy of the model was evaluated through calibration curves, receiver operating characteristic curves (ROC) curves, and decision curves analysis (DCA). The calibration curve was used to determine the consistency between the predicted and actual probabilities, evaluating the fitness of the model. The calibration curves were used to determine the consistency between the predicted and actual probabilities, evaluating the fitness of the model. The discriminative ability of the model was determined using the area under the ROC curve (AUC). DCA revealed the standardized net benefit of the prediction model at different risk thresholds and was used to evaluate its clinical utility. The R packages used in this study are listed in Supplementary Table S2.

### Statistical analysis

Statistical analysis was performed using SPSS 26.0 and R software 4.3.3. All statistical tests were two-tailed, and *P* < 0.05 was considered statistically significant. Normally distributed continuous variables were expressed as means ± standards and were compared between groups using independent samples *t*-test. Non-normally distributed continuous variables were presented using medians (interquartile range) and the Mann-Whitney U test was used for comparison between groups. Frequencies and percentages were used to present categorical variables, and a chi-square test was used to analyze count data. In our study, potential risk factors were first selected using LASSO regression with 10-fold cross-validation, where variables retaining non-zero coefficients under the optimal lambda (selected by minimum binomial deviance) were considered candidates. Multivariable logistic regression was performed to analyze independent risk factors for neonatal severe pneumonia and variables with significant correlations (*p* < 0.05) were selected to develop a nomogram. The final model combined the regularization advantages of LASSO (addressing multicollinearity) with the interpretability of logistic regression.

## Results

### General information and clinical characteristics

**FIGURE 2:**
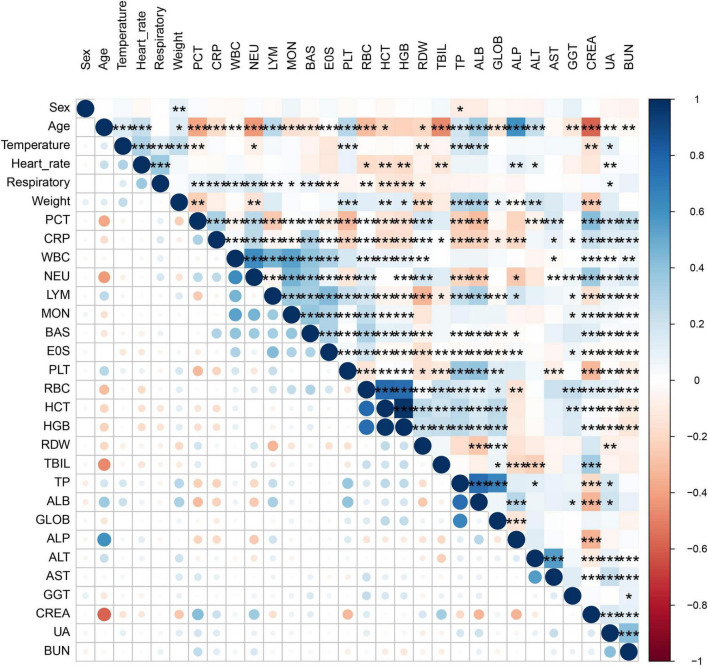
Correlation bubble chart shows the Spearman correlation between parameters. Where one variable is plotted on the x-axis and the other on the y-axis for both severe pneumonia and mild to moderate pneumonia; antique blue for positive correlation and red for negative correlation.

A total of 959 neonates with pneumonia were included in this study. According to the inclusion and exclusion criteria, 652 newborns ultimately participated in the construction of the model. The general information and clinical characteristics of the patients are listed in Supplementary Table S3. The respiratory rate, PCT, CRP, WBC, NEU, MON, RDW, CREA, UA, and BUN were significantly lower in the mild to moderate pneumonia group compared to the severe pneumonia group. Conversely, the mild to moderate pneumonia group exhibited significantly higher levels of age, weight, PLT, HCT, HGB, TP, ALB, GLOB, and ALP compared to the severe pneumonia group. There were no statistically significant differences in gender, heart rate, LYM, EOS, RBC, TBIL, ALT, AST, and GGT levels between the two groups. The difference in temperature and BAS between the two sets of data was not significant. A total of 186 neonates developed severe pneumonia in this study, including 151 cases (81.2%) in the training set and 35 cases (18.8%) in the testing set. There was no significant difference between the training set and testing set for any variables other than GLOB (Table 1). Additionally, we analyzed risk factors for severe pneumonia in the training set. The results indicated that age, temperature, respiratory rate, weight, PCT, CRP, WBC, NEU, LYM, MON, BAS, PLT, HCT, HGB, TP, ALB, ALP, CREA, UA, and BUN were all associated with severe pneumonia. However, there were no significant differences in gender, heart rate, EOS, RBC, RDW, TBIL, GLOB, ALT, AST, and GGT (Table 2). Furthermore, we examined the correlation between various indicators. It was found that some significant indicators were associated with the occurrence of severe pneumonia (Figure 2). We selected indicators with strong correlation through *p*-values and correlation coefficients (*r* values). The heatmap revealed strong collinearity (*r* = 0.97, *p* < 0.001) between HCT and HGB. To avoid the multicollinearity effect, such pairs are intentionally not included simultaneously in the variables screened by LASSO.

**TABLE 1:** Comparison of clinical features of pneumonia patients in the training set and the testing set.

Variables	Total (*n* = 652)	Training set (*n* = 554)	Testing set (*n* = 98)	*P*
Male, *n* (%)	373 (57%)	313 (56%)	60 (61%)	0.447
Age (days)	13 (7, 22)	13 (7, 22)	14 (9, 22)	0.450
Temperature (°C)	36.9 (36.7, 37.1)	36.9 (36.7, 37.1)	37 (36.7, 37.2)	0.460
Heart rate (bpm)	150 (140, 157)	150 (140, 156)	149.50 (140, 159.75)	0.421
Respiratory (rate/minute)	52 (46, 59.25)	52 (46, 59)	55 (47, 63)	0.072
Weight (kg)	3.30 (2.98, 3.72)	3.34 (3.00, 3.75)	3.23 (2.80, 3.66)	0.085
PCT (ng/mL)	0.10 (0.08, 0.18)	0.10 (0.08, 0.17)	0.11 (0.08, 0.20)	0.686
CRP (mg/L)	0.80 (0.50, 0.80)	0.77 (0.50, 0.80)	0.80 (0.50, 0.80)	0.694
WBC (× 10^9^ cells/L)	9.33 (7.58, 11.23)	9.37 (7.54, 11.26)	8.72 (7.71, 10.95)	0.736
NEU (× 10^9^ cells/L)	3.55 (2.45, 5.11)	3.57 (2.51, 5.11)	3.26 (2.15, 5.09)	0.294
LYM (× 10^9^ cells/L)	4.32 (3.17, 5.59)	4.34 (3.14, 5.61)	4.18 (3.33, 5.46)	0.776
MON (× 10^9^ cells/L)	0.80 (0.61, 1.06)	0.81 (0.61, 1.09)	0.76 (0.60, 1.00)	0.179
BAS (× 10^9^ cells/L)	0.03 (0.02, 0.04)	0.03 (0.02, 0.04)	0.03 (0.02, 0.05)	0.490
E0S (× 10^9^ cells/L)	0.31 (0.18, 0.51)	0.31 (0.18, 0.49)	0.29 (0.16, 0.55)	0.865
PLT (× 10^9^ cells/L)	333.00 (240.50, 403.25)	335.00 (238.75, 404.75)	322.00 (247.25, 398.00)	0.610
RBC (× 10^12^ cells/L)	3.95 (3.52, 4.50)	3.96 (3.52, 4.51)	3.86 (3.43, 4.46)	0.238
HCT (%)	38.65 (33.90, 44.00)	38.90 (34.10, 44.08)	37.50 (32.70, 43.40)	0.164
HGB (g/L)	133.00 (117.00, 151.00)	134.00 (117.00, 151.00)	127.50 (112.25, 147.75)	0.144
RDW (%)	14.90 (14.20, 15.60)	14.90 (14.20, 15.60)	14.70 (14.10, 15.60)	0.270
TBIL (μmol/L)	108.05 (47.35, 165.88)	109.40 (47.20, 166.10)	99.45 (50.75, 160.88)	0.762
TP (g/L)	53.95 (49.30, 57.73)	54.10 (49.40, 57.88)	52.85 (49.02, 57.15)	0.116
ALB (U/L)	33.10 (29.28, 36.00)	33.05 (29.33, 36.10)	33.25 (29.02, 35.88)	0.690
GLOB (g/L)	20.70 (18.30, 23.13)	20.80 (18.40, 23.20)	19.85 (17.25, 22.40)	0.031
ALP (U/L)	191.15 (143.20, 243.60)	191.80 (143.35, 242.72)	185.40 (142.68, 244.67)	0.945
ALT (U/L)	28.50 (20.88, 38.40)	28.75 (21.40, 38.77)	26.15 (19.40, 36.97)	0.101
AST (U/L)	35.00 (27.67, 45.52)	35.00 (27.72, 45.40)	35.65 (27.45, 46.65)	0.835
GGT (U/L)	98.50 (67.38, 141.20)	98.25 (66.85, 140.80)	100.15 (71.53, 143.60)	0.626
CREA (mmol/L)	35.70 (27.80, 48.05)	36.00 (27.83, 47.98)	33.80 (27.40, 48.02)	0.540
UA (mmol/L)	140.15 (111.70, 178.02)	142.20 (112.60, 179.57)	134.05 (109.62, 167.33)	0.181
BUN (mmol/L)	2.80 (1.70, 3.70)	2.80 (1.60, 3.70)	2.70 (2.02, 3.70)	0.396

PCT, procalcitonin; CRP, C-reactive protein; WBC, white blood cell; NEU, neutrophils; LYM, lymphocyte; MON, monocyte; BAS, basophil; EOS, eosinophil; PLT, platelet; RBC, red blood cell; HCT, hematocrit; HGB, hemoglobin; RDW, red blood cell distribution width; TBIL, total bilirubin; TP, total Protein; ALB, albumin; GLOB, globulin; ALP, alkaline phosphatase; ALT, alanine aminotransferase; AST, aspartate aminotransferase; GGT, gamma-glutamyl transferase; CREA, creatinine; UA, uric acid; BUN, blood urea nitrogen.

**TABLE 2:** Comparison of clinical characteristics of patients with severe pneumonia and mild to moderate pneumonia in the training set.

Variables	Total (*n* = 554)	Mild to moderate pneumonia (*n* = 403)	Severe pneumonia (*n* = 151)	*P*
Male, *n* (%)	313 (56)	233 (58)	80 (53)	0.354
Age (days)	13 (7, 22)	14 (8.5, 22)	9 (3, 21)	<0.001
Temperature (°C)	36.9 (36.7, 37.1)	37 (36.7, 37.15)	36.9 (36.5, 37)	0.002
Heart rate (bpm)	150 (140, 156)	150 (142, 155.5)	151 (137.5, 158.5)	0.363
Respiratory (rate/minute)	52 (46, 59)	50 (45, 56)	59 (50, 66)	<0.001
Weight (kg)	3.34 (3.00, 3.75)	3.40 (3.10, 3.81)	3.10 (2.69, 3.52)	<0.001
PCT (ng/mL)	0.10 (0.08, 0.17)	0.10 (0.07, 0.13)	0.22 (0.10, 1.34)	<0.001
CRP (mg/L)	0.77 (0.50, 0.80)	0.50 (0.50, 0.80)	0.80 (0.50, 8.62)	<0.001
WBC (× 10^9^ cells/L)	9.37 (7.54, 11.26)	9.17 (7.49, 10.82)	10.15 (7.73, 13.03)	<0.001
NEU (× 10^9^ cells/L)	3.57 (2.51, 5.11)	3.19 (2.32, 4.38)	5.26 (3.58, 7.88)	<0.001
LYM (× 10^9^ cells/L)	4.34 (3.14, 5.61)	4.41 (3.39, 5.56)	3.93 (2.58, 5.73)	0.034
MON (× 10^9^ cells/L)	0.81 (0.61, 1.09)	0.80 (0.61, 1.00)	0.88 (0.64, 1.34)	0.005
BAS (× 10^9^ cells/L)	0.03 (0.02, 0.04)	0.03 (0.02, 0.04)	0.03 (0.02, 0.06)	0.011
E0S (× 10^9^ cells/L)	0.31 (0.18, 0.49)	0.31 (0.20, 0.46)	0.31 (0.10, 0.76)	0.929
PLT (× 10^9^ cells/L)	335.00 (238.75, 404.75)	347.00 (267.00, 410.50)	273.00 (151.50, 373.00)	<0.001
RBC (× 10^12^ cells/L)	3.96 (3.52, 4.51)	4.02 (3.55, 4.50)	3.83 (3.43, 4.54)	0.363
HCT (%)	38.90 (34.10, 44.08)	39.70 (34.85, 44.30)	36.30 (31.20, 42.35)	<0.001
HGB (g/L)	134 (117, 151)	137 (121, 153)	122 (106, 142)	<0.001
RDW (%)	14.90 (14.20, 15.60)	14.90 (14.20, 15.50)	15.20 (14.20, 15.95)	0.067
TBIL (μmol/L)	109.40 (47.20, 166.10)	106.70 (48.40, 164.80)	112.40 (40.40, 168.95)	0.654
TP (g/L)	54.10 (49.40, 57.88)	54.80 (51.00, 58.20)	50.40 (45.20, 56.00)	<0.001
ALB (U/L)	33.05 (29.33, 36.10)	33.90 (31.05, 36.30)	29.80 (26.75, 33.95)	<0.001
GLOB (g/L)	20.80 (18.40, 23.20)	20.80 (18.60, 23.35)	20.60 (17.80, 22.80)	0.159
ALP (U/L)	191.80 (143.35, 242.72)	195.70 (151.15, 247.05)	170.80 (128.20, 223.20)	0.003
ALT (U/L)	28.75 (21.40, 38.77)	28.90 (22.55, 38.65)	27.40 (18.35, 39.05)	0.196
AST (U/L)	35.00 (27.72, 45.40)	35.00 (28.60, 43.75)	34.70 (26.20, 50.25)	0.871
GGT (U/L)	98.25 (66.85, 140.80)	100.30 (67.85, 137.45)	89.90 (61.70, 162.20)	0.646
CREA (mmol/L)	36.00 (27.83, 47.98)	35.00 (27.10, 44.30)	46.10 (32.00, 66.55)	<0.001
UA (mmol/L)	142.20 (112.60, 179.57)	135.00 (111.70, 167.95)	165.20 (116.40, 223.70)	<0.001
BUN (mmol/L)	2.80 (1.60, 3.70)	2.60 (1.50, 3.40)	3.50 (1.95, 4.90)	<0.001

PCT, procalcitonin; CRP, C-reactive protein; WBC, white blood cell; NEU, neutrophils; LYM, lymphocyte; MON, monocyte; BAS, basophil; EOS, eosinophil; PLT, platelet; RBC, red blood cell; HCT, hematocrit; HGB, hemoglobin; RDW, red blood cell distribution width; TBIL, total bilirubin; TP, total Protein; ALB, albumin; GLOB, globulin; ALP, alkaline phosphatase; ALT, alanine aminotransferase; AST, aspartate aminotransferase; GGT, gamma-glutamyl transferase; CREA, creatinine; UA, uric acid; BUN, blood urea nitrogen.

### The construction of nomogram model based on risk factors

**FIGURE 3:**
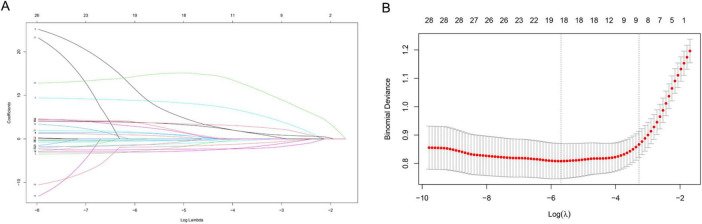
Variable selection using the LASSO binary logistic regression model. **(A)** A coefficient profile was generated based on the logarithmic (lambda) sequence, and the optimal lambda value was used to produce non-zero coefficients. The colored lines represent different variables. As log (λ) gradually increases, the regression coefficient continuously converges until it becomes zero, thereby screening the variables. **(B)** The LASSO coefficient curves for the 30 variables were plotted against the log (λ) sequence. A vertical line was drawn at the value selected using 10-fold cross validation, which resulted in nine non-zero coefficients for the optimal λ. X-axis: Log (λ) value at selection point. Y-axis, Standardized coefficient magnitude.

**FIGURE 4:**
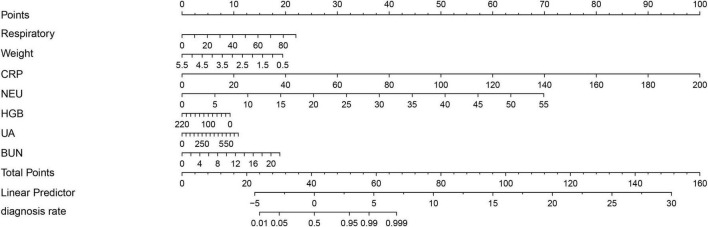
A nomogram has been developed to predict the likelihood of severe pneumonia in neonates with pneumonia. To calculate total score and predicted probability of neonatal severe pneumonia, points from individual variables are added and a vertical line is drawn from the total points line at the bottom downward to determine the diagnosis rate. Example calculation: Estimate the risk for a neonatal severe pneumonia with: respiratory = 40 rate/min (Points: 10), weight = 3 kg (Points: 10), CRP = 150 mg/L (Points: 75), NEU = 10 × 109 cells/L (Points: 12.5), HGB = 0.1 g/L (Points: 10), UA = 250 mmol/L (Points: 3.75), and BUN = 3 mmol/L (Points: 2.5). Step 1, Sum the points (123.75); Step 2, Locate 123.75 on “Total Points” axis; Step 3, Read corresponding risk (>99.9%) on “diagnosis rate” axis.

The LASSO regression analysis was used to identify the best predictors. Multiple logistic regression was used to establish the prediction model. In the training set of 554 neonates with pneumonia including 151 neonates with severe pneumonia, we examined various risk variables. As shown by the vertical dotted line in Figure 3, variables retained when their coefficients became non-zero, that is, the 9 selected predictors corresponded to the last non-zero coefficients before deviance increased by more than 1 SE. By combining clinical experience with the results of the LASSO regression, we identified nine potential predictors with non-zero coefficients (Supplementary Table S4). Further analysis using the multivariate logistic regression revealed that respiratory rate (OR: 1.058, 1.035–1.081), weight (OR: 0.483, 0.340–0.686), CRP (OR: 1.1442, 1.028–1.268), NEU (OR: 1.348, 1.232–1.555), HGB (OR: 0.989, 0.979–0.999), UA (OR: 1.006, 1.002–1.010), and BUN (OR: 1.230, 1.058–1.431) were independent risk factors for severe pneumonia (Supplementary Table S5). We then weighted the regression coefficients of these risk factors included in the regression model and developed a risk score formula for predicting the likelihood of severe pneumonia: Risk score = −2.12 + 0.053 (Respiratory rate) − 0.915 (Weight) + 0.156 (CRP) + 0.349 (NEU) − 0.013 (HGB) + 0.004 (UA) + 0.192 (BUN). Predicted risk = 1/(1 + e − risk score). Based on these above risk factors and regression coefficients, we created a nomogram model for predicting neonatal severe pneumonia (Figure 4). The nomogram revealed the relative contributions of each factor to the risk of neonatal severe pneumonia. We can see that the screened variables predict the probability of neonatal severe pneumonia.

### Validation and evaluation of the nomogram

**FIGURE 5:**
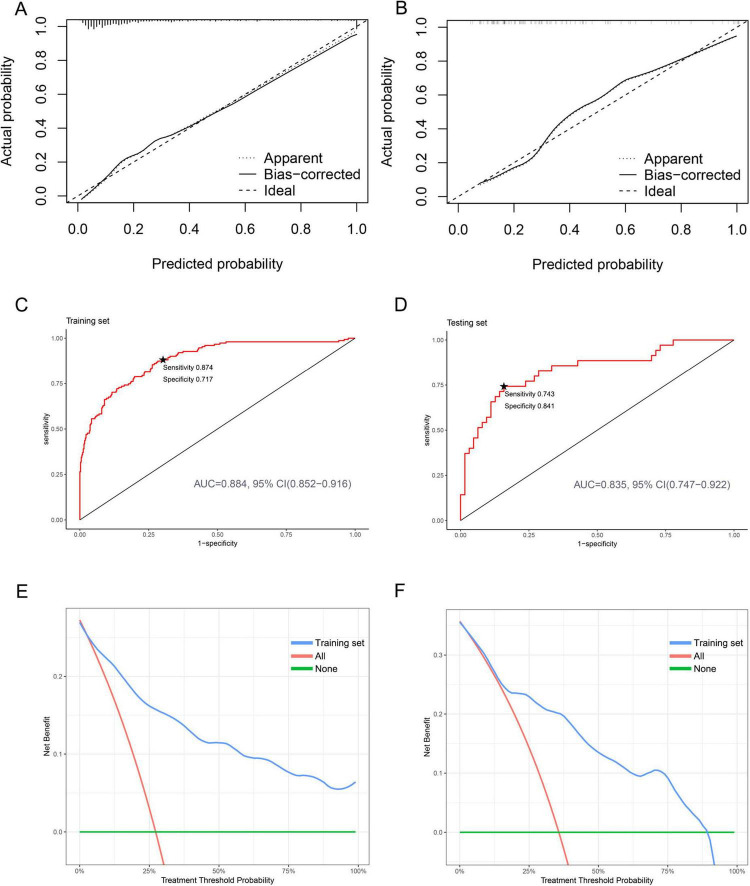
Calibration, discrimination, and clinical application of nomogram prediction models in both the training and testing sets. **(A)** Calibration plot for the training set. **(B)** Calibration plot for the testing set. **(C)** ROC curves for the training set. The starred point (★) denotes the optimal threshold. **(D)** ROC curves for the testing set. The starred point (★) denotes the optimal threshold. **(E)** DCA curves for the training set. **(F)** DCA curves for the testing set.

To assess the fit of our predictive model, we conducted an internal validation of the nomogram. We used calibration curves to compare the predicted probabilities to the actual probabilities. The results showed good alignment between the predicted and actual probabilities in both the training and testing sets (Figures 5A,B). To further evaluate the predictive performance of the nomogram, we analyzed the results using the AUC. Our results showed no significant difference (*P* = 0.302) between the AUC of the training set [AUC: 0.884, 95% CI (0.852–0.916)] and that of the testing set [AUC: 0.835, 95% CI (0.747–0.922)] (Figures 5C,D, Supplementary Table S6). Additionally, we used DCA to evaluate the clinical application value of our predictive model and quantify the net clinical benefit at different thresholds. Our findings showed that the net benefit of our prediction model on the testing set was significantly higher than that of the two extremes, indicating that the nomogram model has better net benefit and prediction accuracy (Figures 5E,F). These results demonstrate that our nomogram has strong discriminatory ability and predictive value, allowing it to accurately identify newborns with mild to moderate pneumonia and severe pneumonia.

## Discussion

The highest risk of death from pneumonia in childhood occurs during the neonatal period. Neonatal pneumonia is a common and serious respiratory illness that is a major cause of infant mortality and morbidity. Chest X-ray remains the first-line imaging method for neonatal pneumonia due to its rapid availability, low cost, and established diagnostic criteria. With the advancement of advanced imaging technology, MRI has shown great value in patients with complex cases that require repeated imaging to evaluate extrapulmonary complications. For example, developing hyperpolarized inhalable gaseous contrast agents for lung imaging (12–14). However, the widespread clinical application of MRI still faces significant challenges and has high technical requirements for the implementers. The majority of negative outcomes from neonatal pneumonia are linked to the development of severe pneumonia. Identifying and treating neonatal severe pneumonia is challenging, as its symptoms are often vague and not specific to the lungs (15). Unlike older children, neonates may not display obvious signs of a localized lung infection, but severe pneumonia typically results in overall deterioration of multiple organ systems (16, 17). It is crucial to accurately identify risk factors for high-risk neonates in order to make informed treatment decisions and effectively reduce mortality rates.

In the past, several scholars have conducted studies on the adverse outcomes of pneumonia in children. In 2016, a large-scale, multicenter, prospective cohort study established a prediction model for serious outcomes in hospitalized children with pneumonia (18). The study identified age extremes, vital signs, chest indrawing, and radiographic infiltrate pattern as the most significant predictors. However, the clinical applicability of this model remains unclear. Another study by Araya et al. developed a scoring system to predict mortality in children hospitalized with community-acquired pneumonia (19). This study found that CRP, PCT, and LYM were the key factors for predicting poor prognosis in severe pneumonia cases. However, it is important to note that there are limited severity scoring systems developed specifically for children with pneumonia. In our study, we analyzed clinical data from neonatal pneumonia cases and established and validated a nomogram model for diagnosing neonatal severe pneumonia. Through LASSO and multivariate logistic regression analysis, we identified respiration rate, weight, CRP, NEU, HGB, UA, and BUN as the main risk factors for neonatal severe pneumonia. Based on these predictors, we developed a nomogram prediction model. After evaluating the performance of the model, we found that it showed good calibration, discrimination, and clinical utility.

Our study showed that rapid respiratory rate was a risk factor for severe pneumonia. The faster the respiratory rate, the higher the risk of developing severe pneumonia. This is consistent with previously reported results. Studies have shown that rapid respiratory rate is a valuable predictor of pneumonia. Respiratory rate > 20/min is an objective indicator of airway resistance due to pneumonia (20). A case-control study found that common risk factors for recurrent pneumonia were preterm birth and respiratory distress at birth (21). Respiratory-related diseases such as wheezing and asthma are also inseparable from the occurrence of pneumonia.

This study has revealed a strong correlation between low birth weight and the occurrence of severe pneumonia, which can significantly worsen the outcomes for affected children. It is widely recognized that newborns, especially those with low birth weight, have a weaker immune system and less developed organs and physiological structure, making them more susceptible to various pathogens (22). Moreover, children with low birth weight have narrower airways, making them more prone to blockages that can hinder their immune response and impair lung function. Research has shown that low birth weight is an independent risk factor for severe pneumonia in hospitalized children (23). Specifically, children with a birth weight of less than 2.5 kg are 5.96 times more likely to develop severe pneumonia compared to those with a birth weight of more than 2.5 kg (24). Furthermore, low birth weight has been linked to a higher risk of death from severe pneumonia (25, 26). A prospective study has also identified low birth weight as a primary risk factor for infection in newborns (27).

This study suggested that CRP was an independent risk factor for neonatal severe pneumonia. CRP, an acute inflammatory factor produced by the liver, plays a critical role in both innate and adaptive immunity. It has been identified as a sensitive and reliable marker of acute inflammation in both infectious and non-infectious diseases. Elevated CRP level is an important indicator for diagnosing pneumonia (28). In children with severe pneumonia, higher serum CRP levels were associated with persistent fever and longer hospital stays (29, 30). Conversely, a decrease in serum CRP levels may indicate a reduction in the inflammatory process, while a persistent increase or fluctuation in CRP levels may suggest ongoing inflammation and a poor prognosis. However, CRP has limited value in the early diagnosis of neonatal infection. Its primary role is to confirm or rule out infection 24 h after clinical suspicion. Previous research has highlighted the significant role of serum CRP levels in the progression and prognosis of pneumonia, which aligns with our findings that CRP can provide valuable predictive insights into the severity of neonatal pneumonia. We hypothesize that the production of high levels of cytokines and chemokines after infection disrupts the innate immune system, attracting inflammatory cells to the lung tissue and causing immune damage. Therefore, CRP is a valuable indicator for predicting the severity and prognosis of neonatal pneumonia.

Our study found a positive correlation between NEU levels and the risk of severe pneumonia. NEU plays a crucial role in initiating and activating inflammatory responses, which serve as the first line of defense against non-specific immune system infections. The recruitment of NEU to the site of inflammation is a characteristic feature of both injuries and acute infections (31, 32). Recent research has highlighted the significant role of NEU in the development of pneumonia. During pneumonia, NEU are recruited to the lungs and work in collaboration with other immune cells to regulate pathogen infection (33). Onishi et al. found that patients with tissue pneumonia who experienced recurrence had elevated the NEU levels in their bronchoalveolar lavage fluid (34). Additionally, studies have shown that critically ill pneumonia patients often exhibit increased NEU and CRP levels, as well as decreased LYM levels, which may be indicative of bacterial infection and compromised cellular immunity. NEU has been identified as an independent risk factor for the deterioration of pneumonia patients’ conditions, highlighting the crucial role of this cell type in the immune system (35). NEU possess a wide range of functions, including chemotaxis, opsonization, phagocytosis, degranulation, and bactericidal action. Through phagocytosis, NEU can effectively kill bacteria within cells. They can also exert antibacterial effects through degranulation. On the one hand, NEU releases effectors such as cationic antimicrobial peptides, serine proteases, myeloperoxidase, and reactive oxygen species (ROS) through degranulation (36). On the other hand, NEU can also release DNA to trap bacteria in neutrophil extracellular traps (NETs) through a process known as NETosis (37). Ultimately, NEU can also produce cytokines to exert anti-inflammatory effects.

This study revealed that HGB serves as a protective factor against neonatal severe pneumonia. HGB is a crucial component of the blood responsible for carrying oxygen, and it also affects blood viscosity and innate immune responses. Previous research has shown that low HGB level is an independent risk factor for increased myocardial energy expenditure in patients with chronic heart failure (38). Additionally, a study by Khalyfa et al. demonstrated that HGB is also protective against coronary heart disease and cancer (39, 40). While HGB has been found to have a negative regulatory role in the development of certain diseases, its role in pneumonia is not yet fully understood. Further research is necessary to determine whether HGB can be used as a diagnostic indicator for severe pneumonia.

Simultaneously, this study also found that UA was a predictor of neonatal severe pneumonia. Serum UA is produced by oxidation of xanthine oxidase in the liver and is a byproduct of purine metabolism, and its levels are regulated by the kidneys. Changes in UA levels can be caused by renal dysfunction or purine metabolism disorders. UA has a variety of biological roles and involved in many pathophysiological processes. Studies have shown that abnormal UA levels are a risk factor for cardiovascular, neurological, and metabolic diseases. For instance, a prospective cohort study found that patients with high UA levels (> 8.5 mg/dl) had a higher risk of all-cause mortality (41). Additionally, Liu et al. found that UA can be used as an indicator of poor prognosis in patients with aspiration pneumonia and is also an independent predictor of refractory *Mycoplasma pneumoniae* pneumonia (42, 43).

This study suggested that BUN was also a predictor of neonatal severe pneumonia. BUN is involved in alterations in renal perfusion and is considered one of the most effective parameters of renal function. According to Peng et al., BUN might help clinicians identify critically ill patients early (44). Recent researchers have also found a relationship between BUN and the occurrence of pneumonia. In fact, BUN has been identified as an important risk factor for ICU admission in patients with COVID-19 (45). Chen observed a significant correlation between elevated BUN levels and poor prognosis in COVID-19 patients (46), which is consistent with our findings. The possible causes for this correlation are as follows: firstly, pathogen infection may directly cause damage to renal tissue; secondly, the deposition of antigen-antibody immune complexes may also contribute to kidney damage; and thirdly, cytokines or inflammatory mediators induced by infection can indirectly affect renal tissue.

In this study, we identified several key risk factors for predicting neonatal severe pneumonia, including respiratory rate, weight, CRP, NEU, HGB, UA, and BUN. The development of this prediction model is a significant achievement, and provides a valuable tool for clinical practitioners to accurately screen newborns for severe pneumonia. This, in turn, can serve as a basis for implementing early prevention and intervention measures. The calibration curve in the graph aligns well with the prediction calibration curve, indicating good performance of the model. The AUC for the training set was 0.884 and for the test set was 0.835, demonstrating strong predictive capabilities. The DCA analysis further confirms the effectiveness of the nomogram prediction model in aiding clinical decision-making for neonatal severe pneumonia. This model holds significant clinical guidance value and has promising application prospects.

This study has several limitations. Firstly, it is a single-center retrospective study, which may introduce some unavoidable biases. Secondly, the research subjects were mainly neonates in Henan Province, and the clinical outcomes of patients under different medical conditions may vary. Whether the model has stable predictive value for other regions remains to be further validated. Given these limitations, future research should focus on multicenter prospective studies, incorporating a broader range of predictive variables, and externally validating the model to enhance its universality and accuracy. Nevertheless, the nomogram model of neonatal severe pneumonia constructed in this study demonstrates good discrimination and calibration capabilities, providing valuable guidance for the early risk prediction of the disease.

## Conclusion

In this study, we developed and validated a prediction model for neonatal severe pneumonia. The model takes into account factors such as respiratory rate, weight, CRP, NEU, HGB, UA, and BUN. By utilizing this model, clinicians can identify high-risk children at an earlier stage and implement timely prevention and intervention measures. This can ultimately lead to improve quality of life and health outcomes for children.

## Data Availability

The data analyzed in this study is subject to the following licenses/restrictions: this study retrospectively included newborns diagnosed with pneumonia who were admitted to the Children’s Hospital Affiliated to Zhengzhou University from January 2019 to December 2022. This research protocol followed the Declaration of Helsinki and was approved by the Ethics Review Board of the Children’s Hospital Affiliated to Zhengzhou University (No. 2022-K-L059). All data in this study was anonymous. The raw data can be obtained from the corresponding author as needed. Requests to access these datasets should be directed to WG, 18838920376@163.com.
