# Supplementary material for: Construction and verification of a risk factor prediction model for neonatal severe pneumonia
Source: Front Med (Lausanne). 2025 Jun 2;12:1536705. doi: 10.3389/fmed.2025.1536705 (PMC12171221; doi:10.3389/fmed.2025.1536705)
Supplement: Supplementary file 1 [file Table_1.docx]

Supplementary Table S1. The definition of pneumonia in children.

| Radiographic | If there is lung or cardiac disease, two serial X-rays demonstrating at least one of the following: |
| --- | --- |
|  | New or progressive infiltrate |
|  | Consolidation |
|  | Cavitation |
|  | Pneumoatocele |
|  | If there is no lung or cardiac disease, one definitive imaging test result is acceptable |
| Worsening gas exchange | Any of the following: |
|  | O_2_ desaturation |
|  | Increased oxygen requirement |
|  | Increased ventilator demand |
| Clinical/laboratory evidence | Must have at least three of the following: |
|  | Temperature instability |
|  | Leukopenia (≤4000 WBC/mm^3^) or leukocytosis (≥15,000 WBC/mm^3^) and left shift (≥10% band forms) |
|  | New onset of purulent sputum or change in character of sputum, or increased respiratory secretions or increased suctioning requirements |
|  | Apnea, tachypnea, nasal flaring with retractions of the chest wall or nasal flaring with grunting |
|  | Wheezing, rales, or rhonchi |
|  | Cough |
|  | Bradycardia (<100 beats/min) or tachycardia (>170 beats/min) |
