# Supplementary material for: Construction and verification of a risk factor prediction model for neonatal severe pneumonia
Source: Front Med (Lausanne). 2025 Jun 2;12:1536705. doi: 10.3389/fmed.2025.1536705 (PMC12171221; doi:10.3389/fmed.2025.1536705)
Supplement: Supplementary file 2 [file Table_2.docx]

Supplementary Table S2. The R packages used in our study.

| Packages | Analysis |
| --- | --- |
| corrplot | Calculating correlation matrices |
| glmnet, and car | Least absolute shrinkage and selection operator (LASSO) regression |
| rms | Nomograms and calibration curves |
| pROC, and ggplot2 | Receiver operating characteristic curve |
| dcurves | Decision curve analysis and clinical impact curve |
