# Supplementary material for: Construction and verification of a risk factor prediction model for neonatal severe pneumonia
Source: Front Med (Lausanne). 2025 Jun 2;12:1536705. doi: 10.3389/fmed.2025.1536705 (PMC12171221; doi:10.3389/fmed.2025.1536705)
Supplement: Supplementary file 3 [file Table_3.docx]

Supplementary Table S3. Baseline characteristics of the studied participants.

| Variables | Total (n = 652) | Mild to moderate pneumonia (n = 466) | Severe pneumonia (n = 186) | *P* |
| --- | --- | --- | --- | --- |
| Male, n (%) | 373 (57%) | 268 (58%) | 105 (56%) | 0.874 |
| Age (days) | 13 (7, 22) | 14 (9, 22) | 9 (3, 21) | < 0.001 |
| Temperature (°C) | 36.9 (36.7, 37.1) | 37 (36.7, 37.1) | 36.9 (36.5, 37.1) | 0.003 |
| Heart rate (bpm) | 150 (140, 157) | 150 (141, 156) | 152 (138, 160) | 0.152 |
| Respiratory (rate/minute) | 52 (46, 59) | 50 (45, 57) | 59 (52, 67) | < 0.001 |
| Weight (kg) | 3.30 (2.98, 3.72) | 3.40 (3.06, 3.81) | 3.08 (2.69, 3.50) | < 0.001 |
| PCT (ng/mL) | 0.10 (0.08, 0.18) | 0.10 (0.07, 0.13) | 0.20 (0.10, 1.17) | < 0.001 |
| CRP (mg/L) | 0.80 (0.50, 0.80) | 0.50 (0.50, 0.80) | 0.80 (0.50, 8.62) | < 0.001 |
| WBC (×10^9^ cells/L) | 9.33 (7.58, 11.23) | 8.93 (7.46, 10.77) | 10.22 (7.79, 13.17) | < 0.001 |
| NEU (×10^9^ cells/L) | 3.55 (2.45, 5.11) | 3.16 (2.26, 4.29) | 5.26 (3.42, 7.89) | < 0.001 |
| LYM (×10^9^ cells/L) | 4.32 (3.17, 5.59) | 4.36 (3.39, 5.49) | 4.06 (2.63, 5.80) | 0.105 |
| MON (×10^9^ cells/L) | 0.80 (0.61, 1.06) | 0.78 (0.61, 1.00) | 0.88 (0.63, 1.32) | 0.001 |
| BAS (×10^9^ cells/L) | 0.03 (0.02, 0.04) | 0.03 (0.02, 0.04) | 0.03 (0.02, 0.06) | 0.001 |
| E0S (×10^9^ cells/L) | 0.31 (0.18, 0.51) | 0.31 (0.19, 0.47) | 0.31 (0.10, 0.74) | 0.873 |
| PLT (×10^9^ cells/L) | 333.00 (240.50, 403.25) | 347.00 (270.00, 410.00) | 270.00 (159.25, 368.25) | < 0.001 |
| RBC (×10^12^ cells/L) | 3.95 (3.52, 4.50) | 4.01 (3.57, 4.49) | 3.80 (3.34, 4.52) | 0.131 |
| HCT (%) | 38.65 (33.90, 44.00) | 39.60 (34.90, 44.30) | 36.30 (30.95, 41.85) | < 0.001 |
| HGB (g/L) | 133.00 (117.00, 151.00) | 136.50 (121.00, 152.75) | 122.00 (106.00, 140.00) | < 0.001 |
| RDW (%) | 14.90 (14.20, 15.60) | 14.80 (14.20, 15.50) | 15.20 (14.10, 16.00) | 0.047 |
| TBIL (μmol/L) | 108.05 (47.35, 165.88) | 107.05 (50.15, 165.33) | 109.45 (40.25, 165.57) | 0.393 |
| TP (g/L) | 53.95 (49.30, 57.73) | 54.65 (51.05, 58.20) | 50.25 (45.20, 55.77) | < 0.001 |
| ALB (U/L) | 33.10 (29.28, 36.00) | 33.95 (31.13, 36.30) | 29.80 (26.40, 34.27) | < 0.001 |
| GLOB (g/L) | 20.70 (18.30, 23.13) | 20.80 (18.60, 23.30) | 20.30 (17.42, 22.50) | 0.032 |
| ALP (U/L) | 191.15 (143.20, 243.60) | 195.20 (151.40, 247.92) | 169.05 (128.38, 226.38) | < 0.001 |
| ALT (U/L) | 28.50 (20.88, 38.40) | 28.75 (22.00, 38.18) | 27.35 (18.12, 39.55) | 0.280 |
| AST (U/L) | 35.00 (27.67, 45.52) | 35.00 (28.45, 43.82) | 35.05 (26.20, 50.27) | 0.756 |
| GGT (U/L) | 98.50 (67.38, 141.20) | 100.20 (68.65, 137.05) | 92.90 (61.88, 166.32) | 0.708 |
| CREA (mmol/L) | 35.70 (27.80, 48.05) | 34.60 (27.02, 44.13) | 46.00 (30.10, 65.85) | < 0.001 |
| UA (mmol/L) | 140.15 (111.70, 178.02) | 135.05 (110.55, 167.10) | 162.45 (114.08, 219.23) | < 0.001 |
| BUN (mmol/L) | 2.80 (1.70, 3.70) | 2.60 (1.60, 3.40) | 3.25 (2.00, 4.90) | < 0.001 |

Abbreviations: PCT: procalcitonin; CRP: C-reactive protein; WBC: white blood cell; NEU: neutrophils; LYM: lymphocyte; MON: monocyte; BAS: basophil; EOS: eosinophil; PLT: platelet; RBC: red blood cell; HCT: hematocrit; HGB: hemoglobin; RDW: red blood cell distribution width; TBIL: total bilirubin; TP: total Protein; ALB: albumin; GLOB: globulin; ALP: alkaline phosphatase; ALT: alanine aminotransferase; AST: aspartate aminotransferase; GGT: gamma-glutamyl transferase; CREA: creatinine; UA: uric acid; BUN: blood urea nitrogen.
