# Supplementary material for: Construction and verification of a risk factor prediction model for neonatal severe pneumonia
Source: Front Med (Lausanne). 2025 Jun 2;12:1536705. doi: 10.3389/fmed.2025.1536705 (PMC12171221; doi:10.3389/fmed.2025.1536705)
Supplement: Supplementary file 4 [file Table_4.docx]

Supplementary Table S4. Coefficients and lambda.1SE value of the LASSO regression.

| Variables | Coefficients | lambda.1SE | Log (Lambda) |
| --- | --- | --- | --- |
| Intercept | -0.91673834 | 0.03785167 | -2.47 |
| Respiratory | 4.90283878 |  |  |
| Weight | -1.45885126 |  |  |
| CRP | 1.06678002 |  |  |
| NEU | 10.31571231 |  |  |
| HGB | -0.82615693 |  |  |
| TP | -0.07789354 |  |  |
| ALB | -2.04559411 |  |  |
| UA | 0.72392763 |  |  |
| BUN | 2.5006666 |  |  |
| Abbreviations: CRP: C-reactive protein; NEU: neutrophils; HGB: hemoglobin; TP: total Protein; ALB: albumin; UA: uric acid; BUN: blood urea nitrogen | | | |
