# Supplementary material for: Construction and verification of a risk factor prediction model for neonatal severe pneumonia
Source: Front Med (Lausanne). 2025 Jun 2;12:1536705. doi: 10.3389/fmed.2025.1536705 (PMC12171221; doi:10.3389/fmed.2025.1536705)
Supplement: Supplementary file 5 [file Table_5.docx]

Supplementary Table S5. Association between risk factors and severe pneumonia in multivariate logistic regression.

| Variables | *β* | OR (95% CI) | *P* |
| --- | --- | --- | --- |
| Intercept | -2.12 |  | 0.031 |
| Respiratory | 0.053 | 1.058 (1.035 - 1.081) | <0.001 |
| Weight | -0.915 | 0.483 (0.340 - 0.686) | <0.001 |
| CRP | 0.156 | 1.142 (1.028 - 1.268) | 0.007 |
| NEU | 0.349 | 1.384 (1.232 - 1.555) | <0.001 |
| HGB | -0.013 | 0.989 (0.979 - 0.999) | 0.008 |
| UA | 0.004 | 1.006 (1.002 - 1.010) | 0.044 |
| BUN | 0.192 | 1.230 (1.058 - 1.431) | 0.012 |
| Abbreviations: CRP: C-reactive protein; NEU: neutrophils; HGB: hemoglobin; UA: uric acid; BUN: blood urea nitrogen | | | |
