# Supplementary material for: Construction and verification of a risk factor prediction model for neonatal severe pneumonia
Source: Front Med (Lausanne). 2025 Jun 2;12:1536705. doi: 10.3389/fmed.2025.1536705 (PMC12171221; doi:10.3389/fmed.2025.1536705)
Supplement: Supplementary file 6 [file Table_6.docx]

Supplementary Table S6. Diagnostic performance of a nomogram model for severe pneumonia in the training set and testing set.

| Variable | Value | |
| --- | --- | --- |
|  | Training set | Testing set |
| AUC | 0.884 | 0.835 |
| 95% CI | 0.852-0.916 | 0.747-0.922 |
| Sensitivity | 0.874 | 0.743 |
| Specificity | 0.717 | 0.841 |
